# An Obesity-Related *FTO* Variant and the Risk of Preeclampsia in a Finnish Study Population

**DOI:** 10.1155/2011/251470

**Published:** 2011-11-03

**Authors:** Miira Klemetti, Leena M. Hiltunen, Sanna Heino, Seppo Heinonen, Eero Kajantie, Hannele Laivuori

**Affiliations:** ^1^Department of Obstetrics and Gynecology, Helsinki University Central Hospital, P.O. Box 140, 00029 Helsinki, Finland; ^2^Department of Medical Genetics, Haartman Institute, Biomedicum Helsinki, University of Helsinki, P.O. Box 63, 00014 Helsinki, Finland; ^3^Women's Health Research Program, University of Helsinki, Helsinki, Finland; ^4^Department of Hemostasis, Finnish Red Cross Blood Service, Kivihaantie 7, 00310 Helsinki, Finland; ^5^Department of Obstetrics and Gynecology, Kuopio University Hospital, P.O. Box 1777, 70211 Kuopio, Finland; ^6^Diabetes Prevention Unit, National Institute of Health and Welfare, P.O. Box 30, 00271 Helsinki, Finland; ^7^Hospital for Children and Adolescents, Helsinki University Central Hospital, P.O. Box 281, 00029 Helsinki, Finland

## Abstract

Previous studies have demonstrated a common variant of the obesity and fat mass-related *FTO* gene, rs9939609, to be associated with obesity, type 2 diabetes, and elevated blood pressure. We investigated whether the *FTO* SNP rs9939609 is associated with the risk of preeclampsia (PE) in a Finnish study population. 485 women with prior PE and 449 women who had given birth after a normotensive pregnancy were genotyped (TaqMan) for the SNP rs9939609. The prevalences of genotypes AA, AT, and TT were 15%, 53%, and 32%, respectively, among the PE cases, and 16%, 47%, and 37%, respectively, among the controls (*P* = 0.199). We found no evidence of an association between the *FTO* SNP rs9939609 and PE. However, our cases were dominated by severe, early-onset PE. Thus, we are unable to exclude an association with the milder, later-onset form of the disease in which the role of maternal metabolic predisposition could be more significant.

## 1. Introduction

Along with the obesity pandemic, women of reproductive age are gaining weight [1]. Overweight and obesity predispose to various pregnancy complications, such as preeclampsia (PE) [2]. PE is a potentially life-threatening syndrome of hypertension, proteinuria, generalized vasoconstriction, and platelet consumption, complicating 2–6% of first pregnancies [3]. Its exact pathogenesis remains unclear, but both genetic and environmental factors seem to play a role. Studies have shown links between the pathologic characteristics of the metabolic syndrome, gestational diabetes and type 2 diabetes (T2DM), and those of PE (e.g., obesity, hyperinsulinemia, hypertriglyceridemia, thrombotic and proinflammatory changes, endothelial dysfunction, and sympathetic overactivity) [4–6]. Many of the metabolic abnormalities appear to persist several years postpartum [7–10]. In line with these findings, patients with prior PE seem to be at elevated risk of developing impaired glucose tolerance or T2DM in later life [11, 12].

Genome-wide association studies in several ethnic populations have shown a common variant of the highly polymorphic obesity and fat mass-related *FTO* gene, rs9939609 (T/A), located on chromosome 16q2, to be associated with obesity and T2DM [13, 14]. While the mechanisms of this association are not known, the effect of *FTO* variants on the risk of T2DM is mediated by the effect of *FTO* on the body-mass index (BMI). Recent studies have revealed linkages between *FTO *variants and gestational diabetes [15], the polycystic ovary syndrome (PCOS) [16] as well as the metabolic syndrome and hyperandrogenism in PCOS women [17, 18]. The A-allele of rs9939689 has also been associated with elevated blood pressure in Finnish women and French Canadians [19, 20]. As PE is linked to many of the above-mentioned conditions associated with *FTO* variants, we decided to investigate whether the SNP rs9939609 is associated with the risk of PE in a Finnish study population.

## 2. Material and Methods

We genotyped the *FTO* SNP rs9939609 in 485 Finnish women with prior PE in a singleton pregnancy and 449 control women who had given birth after a normotensive singleton pregnancy. The subjects came from three different studies described below. All subjects provided a written informed consent, and all study protocols were approved by the appropriate local ethical committees. In addition, the approval of the Finnish Ministry of Social Affairs and Health was obtained for the population-based study (data set 1). 

The diagnostic criteria of PE used are defined below separately for each study. Systolic blood pressure ≥ 160 mmHg and/or diastolic blood pressure ≥ 110 mmHg was classified as severe PE. The criteria for severe PE used in this study do not include the severity of proteinuria as quantitative measurements of proteinuria were not available for all cases. 

Prepregnancy weight and height were obtained from the antenatal care records, where they are in most cases recorded as reported by the mother at the first maternity clinic visit, usually before 12 weeks of gestation. Body mass index (BMI) was defined as the prepregnancy weight in kilograms divided by height in meters squared (kg/m^2^). BMI  ≥  25 kg/m^2^ was classified as overweight and BMI  ≥  30 kg/m^2^ as obese. Small-for-gestational age (SGA) was defined as relative birth weight under −2.0 SD units (*Z* score) according to Finnish standards [21].

### 2.1. Data Set 1: Finnish Population-Based Preeclampsia Study

The cases and controls for the population-based data set were identified by combining two national registers [22]. In Finland, pregnant women are registered in the National Register of Blood Group and Blood Group Antibodies of Pregnant Women at the Finnish Red Cross Blood Service, from which 100.000 consecutive pregnant women were identified during 1997-1998. Of these, 1084 had an International Classification of Diseases (ICD-10) code for PE or eclampsia in the National Hospital Discharge Register maintained by the National Research and Development Centre for Welfare and Health. First, 665 women with PE diagnosis were invited to the study, and 411 (62%) participated. After checking the case records and excluding multiple pregnancies, 226 cases with singleton pregnancies fulfilled the criteria for PE. In addition to PE patients, a random sample of women without pregnancy complications (*n* = 1930) were invited to the study, and 843 (44%) participated. Of these, 346 women with singleton full-term pregnancies without hypertensive complications, matched as closely as possible with the cases for the province of residence, parity, and maternal age, served as controls in this study. All participants were of Finnish ethnic origin, gave blood samples for the study, and filled out questionnaires to supplement the data obtained from medical records.

PE was defined as systolic blood pressure ≥ 140 mmHg and diastolic blood pressure ≥ 90 mmHg with new-onset proteinuria (0.3 g/l or ≥0.5 g/24 hours or dipstick ≥ + representing values ≥0.3 g/l) after 20 weeks of gestation in a previously normotensive woman (slightly modified from ACOG criteria) [22, 23]. The highest blood pressure values were recorded.

### 2.2. Data Set 2: Southern Finland Preeclampsia Study

Using the discharge records, we identified women who had developed severe PE and given birth in Helsinki University Central Hospital between January 1988 and April 1998 [24]. Patients with multiple pregnancies were excluded. Blood samples were collected between January 1997 and April 1998 after the index pregnancy. During the same period, blood samples were collected from control subjects with singleton deliveries in the same hospital after uncomplicated pregnancies. In total, 129 preeclamptic women and 103 normotensive healthy controls with no pregnancy complications were recruited. Among the PE cases, 102 women had a systolic blood pressure ≥160 mmHg and/or a diastolic blood pressure ≥110 mmHg, and 61 patients had proteinuria of at least 5 g per 24-hour urine collection. All subjects were of Finnish ethnic origin, lived in southern Finland, and had been healthy before their first pregnancy, without evidence of renal or autoimmune diseases. At 12 weeks postpartum, all women were normotensive, and proteinuria had disappeared. 

The diagnostic criteria of PE used were systolic blood pressure ≥ 140 mmHg and diastolic blood pressure ≥ 90 mmHg with new-onset proteinuria (≥0.3 g/24 hours) after 20 weeks of gestation in a previously normotensive woman [24]. The blood pressure was confirmed by two measurements taken at least 6 hours apart.

### 2.3. Data Set 3: Eastern Finland Preeclampsia Study

The samples of this data set were collected retrospectively from women with prior PE when primiparous who delivered at Kuopio University Hospital between January 1994 and December 1998 [25]. PE patients were identified from the Birth Registry of the City of Kuopio, contacted by telephone, and asked to participate in the study and sign a consent form. Patients with multiple pregnancies were excluded. In total, samples obtained from 130 women with prior PE in a singleton pregnancy were analyzed. 

In this study, PE was defined as the development of hypertension and new-onset proteinuria (≥300 mg of urinary protein per 24 hours). Hypertension was defined as ≥140 mmHg systolic and/or ≥90 mmHg diastolic pressure, when measured on two consecutive occasions at least 24 hours apart [26]. Women with essential hypertension were excluded from the study.

### 2.4. SNP Genotyping

The *FTO* SNP (rs9939609) was genotyped using an ABI TaqMan allele discrimination predesigned SNP genotyping assay (Applied Biosystems). Polymerase chain reaction (PCR) amplification was carried out according to the manufacturer's instructions. An ABI 7500 real-time thermocycler (Applied Biosystems) was used to perform plate reading. Automated allele calling was performed by allelic discrimination plots using Applied Biosystems 7500/7500 fast real-time PCR Software v.2.0.

### 2.5. Statistical Analyses

The Hardy-Weinberg equilibrium calculator of the Genetic Online Encyclopedia (http://www.oege.org/software/hardy-weinberg.shtml) was used to test for deviations in genotype distributions from the Hardy-Weinberg equilibrium. Power analysis was performed assuming a PE prevalence of 3%, high-risk allele (A) frequency of 0.3272 [27], genotypic relative risk of 1.5 for AT, and 2.0 for AA. With these assumptions, a sample size of 263 PE cases was estimated to result in a power >0.80 when *α* < 0.05.

Differences of the background characteristics between cases and controls were analyzed using the Student's *t*-test or the Mann-Whitney test (continuous variables) and the Chi-square test or the Fisher's exact test (frequencies for discrete variables). Of the continuous variables, the BMI had a right-skewed distribution, and the birth weight in grams had a left-skewed distribution. The BMI was log transformed to attain normality for the logistic regression analyses of Table 3. Differences in the frequencies of genotypes and alleles were analyzed using the Chi-square test. Logistic regression analysis was used for the calculation of crude and adjusted odds ratios (OR). Linear regression analysis was used to determine the association of continuous variables with the risk allele. One-way analysis of variance (ANOVA) and the Bonferroni post hoc test were used to compare the means of continuous variables among the different genotype groups and data sets. *P* value < 0.05 was considered statistically significant in all analyses. The statistical software used was PASW Statistics 18.0 and Prism for Windows, version 4.03, GrapPad Software Inc, La Jolla, Calif, USA.

## 3. Results

### 3.1. Characteristics of the Study Subjects

The combined background characteristics of the three study populations used in this study are presented in Table 1. There was no significant difference in the mean age of PE cases and controls. Expectably, the cases had a higher mean BMI than the controls although the difference was statistically significant only among the primipara. In addition, the cases delivered earlier and their infants had a lower birth weight. Pregestational and gestational diabetes were slightly more common among the primiparous PE cases than among the primiparous control women.

When the PE cases of the three data sets were studied separately, significant differences in the background characteristics and risk factors were found between the data sets. The mean BMI values among the PE cases in data sets 1, 2, and 3 were 24.3 kg/m^2^, 22.4 kg/m^2^, and 24.8 kg/m^2^, respectively, with the difference between data set 2 and the other two data sets being statistically significant (*P* < 0.001). In data sets 1 and 2, the mean systolic blood pressure used to define PE in cases was similar, 170.2 mmHg and 170.9 mmHg, respectively, but in data set 3, it was significantly lower, 161.0 mmHg (*P* < 0.001). Correspondingly, the mean diastolic blood pressure values used to define PE in data sets 1 and 2 were 106.1 mmHg and 107.2 mmHg, but in data set 3, the mean was 102.9 mmHg (*P*  =  0.016 and *P*  =  0.002 for data set 3 versus data sets 1 and 2, resp.). The mean gestational age at delivery was higher among the PE cases of data set 1 (36.6 weeks gestation) compared with those of data sets 2 (34.4 weeks gestation) and 3 (35.1 weeks gestation) (*P* < 0.001 and *P* = 0.001, resp.). Also the relative birth weight was higher among the offspring of cases of data set 1 (−1,0 SD units) compared with those of data set 2 (−1.6 SD units) and data set 3 (−1.4 SD units) (*P* = 0.001 and 0.017, resp.).

### 3.2. Risk Factors of Preeclampsia

Well-established risk factors of PE such as BMI (odds ratio [OR] for each kg/m^2^ increase in BMI = 1.07; 95% CI: 1.04, 1.11; *P* < 0.001), primiparity (OR  =  1.37; 95% CI: 1.05, 1.79; *P* = 0.020), and pregestational diabetes (OR = 6.16; 95% CI: 1.38, 27.4; *P* = 0.017) were associated PE also in this study population when all three data sets were combined. When the data sets were analyzed separately, prepregnancy BMI was associated with PE (OR = 1.11; 95% CI: 1.06–1.15; *P* < 0.001) and severe PE defined by hypertensive criteria only (OR: 1.10; 95% CI: 1.06–1.16; *P* < 0.001) in data set 1 but not in data set 2 (OR  =  0.95; 95% CI: 0.87, 1.03; *P* = 0.172; and OR = 0.963; 95% CI: 0.89, 1.05; *P* = 0.963, resp.).

Overall, 17.9% of all study subjects were overweight and 7.5% were obese. In data sets 1, 2, and 3, the percentages of overweight and obese patients were 19.1% and 7.0%, 12.1% and 4.3%, and 23.1% and 15.4%, respectively. No difference in the frequency of overweight or obesity was found between subjects homozygous for the A-allele of rs9939609 and those homozygous for the T-allele (*P* = 0.406 and *P* = 0.134, resp.). In the entire study population, each additional A-allele corresponded to an increase in BMI of 0.145 kg/m^2^, but the result was statistically nonsignificant (95% CI: −0.245, 0.535, *P* = 0.446). Among the cases and the controls, the BMI effect per one A-allele was 0.082 kg/m^2^ (*P* = 0.791, 95% CI: −0.526, 0.690) and 0.025 kg/m^2^ (*P* = 0.923, 95% CI: −0.474, 0.523), respectively. In data sets 1, 2, and 3, the BMI-changes per each additional A-allele were 0.197 (95% CI: −0.291, 0.685; *P* = 0.427), −0.412 (95% CI: −1.043, 0.219, *P* = 0.200), and 1.409 (95% CI: −0.034, 2.852; *P* = 0.056), respectively. The mean BMI for the genotypes TT, AT, and AA were 23.2, 23.5, and 23.5 for data set 1, 23.1, 22.5, and 22.3 for data set 2 and 24.2, 24.9, and 27.5 for data set 3, respectively, with all differences between groups being statistically nonsignificant.

### 3.3. rs9939609 and Preeclampsia

The rs9939609 genotypes and allele frequencies among the PE cases and controls are presented in Table 2. The genotype distributions did not deviate from the Hardy-Weinberg equilibrium among the cases or the controls. No evidence of an association between SNP rs9939609 of *FTO* and PE was found in this study. The prevalences of genotypes AA, AT, and TT were 15%, 53%, and 32%, respectively, among the PE cases, and 16%, 47%, and 37%, respectively, among the controls (*P* = 0.199). The crude and adjusted odds ratios for the association of *FTO *with PE, severe PE, and the delivery of a small-for-gestational-age (SGA) infant are presented in Table 3.

## 4. Discussion

Although overweight is a major risk factor of pregnancy-induced hypertension and PE [2], we were unable to demonstrate an association between the obesity-predisposing rs9939609 of *FTO *and PE in this study. To the best of our knowledge, no reports on this subject have been published previously. 

The study population used in this study can be considered fairly representative of the Finnish population as it is composed of a population-based sample as well as subjects recruited from hospitals with geographically based catchment areas in both Eastern and Southern Finland. The A-allele frequency among this study population is somewhat higher than that reported by another Finnish study [27] but in line with the frequency demonstrated in other European populations [28]. 

One of the weaknesses of the data sets used in this study was the lack of information on lifestyle factors, which could have influenced the association of rs9939609 with BMI. Past studies regarding the interaction of physical activity and the effect of the *FTO* genotype on BMI have been somewhat conflicting. Karasawa et al. [29] studied the effect of energy expenditure for moderate to high-intensity physical activity on the association of rs9939609 of the *FTO* with obesity. The group found BMI to be significantly higher and obesity more frequent in the group with genotype AA and low physical activity, but not in the group with genotype AA and high physical activity. Other researchers have also reported the effect of *FTO* on obesity to be exacerbated by low physical activity [30–33]. On the contrary, a study by Jonsson et al. [34] with a sample of 15925 Swedish and 2511 Finnish nondiabetic adults did not support the notion that physical activity modifies the effects of the *FTO* rs9939609 variant on obesity. Furthermore, Lappalainen et al. [35] found no association between *FTO *variant and the magnitude of weight reduction achieved by a long-term lifestyle intervention in the Finnish Diabetes Prevention Study (DPS) among middle-aged subjects with impaired glucose tolerance.

The study population consisted entirely of women of reproductive age. However, the lack of male study subjects should not be behind the lack of BMI association as previous studies have consistently shown the A-allele of rs9939609 to predispose to obesity independent of sex [13]. The few studies that have reported sex differences have demonstrated a stronger effect among women [35, 36]. It is unlikely that the mean age of the study populations would conceal the BMI-effect because the association of the rs9939609 of *FTO* with BMI has been clearly shown among both children and adults [13, 28]. Hardy et al. [37] have, however, demonstrated the effect to peak at 20 years and then weaken with progressing age. 

The present study population was relatively lean, with the mean BMIs ranging from 22.4 to 24.8 in the three data sets analysed. However, the prepregnancy body weight was self-reported by the study subjects and thus might be somewhat underreported. Nevertheless, the leanness of the study population might be one factor contributing to the lack of association of *FTO* with BMI in this study. This is supported by the fact that the *FTO*-BMI effect per one A-allele was the largest and approached statistical significance among the subjects of data set 3, which included more overweight and obese subjects than the other data sets. The results of Jacobsson et al. [33] also suggested that the impact of *FTO* on obesity may be less prominent in lean populations. On the other hand, the *FTO*-BMI association was clear in the ALSPAC study population, which was composed of women of reproductive age with a mean BMI comparable to the one reported in the present study [28].

The differences in the background characteristics of the three PE data sets could explain why no association was demonstrated between the rs9939609 of *FTO* and BMI or PE in this study. The PE cases in data set 2 suffered predominantly from the severe, earlier-onset form of PE with high proteinuria, whereas in data sets 1 and 3, the severity of PE among the cases varied. In data set 2, the cases were the leanest, BMI was not associated with an increased risk of PE, and the gestational age at delivery was the lowest. This is in line with the hypothesis that the early-onset PE is characterized by abnormal placentation and a stronger genetic component, while the role of maternal metabolic predisposition is more significant in the late-onset form of the disease [38].

## 5. Conclusions

We found no evidence of an association between the fat-mass-associated SNP rs9939609 and PE. It is possible that risk factors other than obesity dominate among patients manifesting severe, early-onset PE compared to those with a milder, later-onset form of the disease. Our cases were dominated by the more severe end of the disease spectrum, and therefore we are unable to exclude an association in particular with the less severe, later-onset forms of the disease. The possible association of the fat-mass-associated SNP rs9939609 and PE should be further investigated with larger sample sizes and with possibilities of stratifying the sample according to the degree of severity and the gestation weeks at the onset of the disease.

## Figures and Tables

**Table 1:** Background characteristics of the Finnish preeclampsia patients and control subjects from data sets 1, 2, and 3.

	Preeclampsia						Controls							
	Primiparous (*n* = 321)			Multiparous (*n* = 164)			Primiparous (*n* = 264)			Multiparous (*n* = 185)			Primiparous	Multiparous
	*N*	Mean	SD	*N*	Mean	SD	*N*	Mean	SD	*N*	Mean	SD	P*	P*
Age (years)	321	28.0	5.4	164	31.8	5.2	264	27.6	4.7	185	31.0	4.9	0.381	0.177
BMI (kg/m^2^)	309	23.7	4.4	163	24.4	4.4	261	22.4	3.3	183	23.4	3.7	<0.001	0.055
Systolic BP (mmHg)	315	167.0	18.4	154	169.6	17.1	80	118.6	9.0	22	119.1	11.9	<0.001	<0.001
Diastolic BP (mmHg)	316	105.7	10.1	154	105.1	10.4	80	73.6	7.8	22	72.2	8.9	<0.001	<0.001
Birth weight (g)	320	2400	934.6	162	2382.6	863.2	264	3537.8	404.0	185	3635.0	412.8	<0.001	<0.001
Relative birth weight (SD)	311	−1.3	1.3	160	−1.2	1.3	262	−0.2	0.8	183	0.0	0.8	<0.001	<0.001
Gestational age at birth (weeks)	315	35.7	3.9	164	35.5	3.5	264	39.9	1.5	185	40.0	1.2	<0.001	<0.001

	*N*	%		*N*	%		*N*	%		*N*	%		P**	P**

Gestational diabetes	16	5.0		7	4.3		5	1.9		12	6.5		0.046	0.362
Pregestational diabetes	8	2.5		5	3.0		1	0.4		1	0.5		0.046	0.103

*Student's *t*-test or Mann-Whitney test. **Chi-square test or Fisher's exact test.

**Table 2:** *FTO* SNP rs9939609 genotypes and allele counts among the Finnish preeclampsia patients and control subjects from data sets 1, 2, and 3.

	Preeclampsia			Controls			P**		
	Primiparous	Multiparous	Combined	Primiparous	Multiparous	Combined	Primiparous	Multiparous	Combined
	*n* = 321	*n* = 164	*n* = 485	*n* = 264	*n* = 185	*n* = 449			
Genotypes									
AA	51	20	71	39	30	69			
AT	159	92	251	118	92	210			
TT	106	48	154	104	63	167	0.291	0.328	0.199
N/A*	5	4	9	3	0	3			
Alleles (frequency)									
A	261 (0.41)	132 (0.41)	393 (0.41)	196 (0.38)	152 (0.41)	348 (0.39)			
T	371 (0.59)	188 (0.59)	559 (0.59)	326 (0.62)	218 (0.59)	544 (0.61)	0.195	0.964	0.321

*Genotyping failed.

**Chi-square test.

**Table 3:** The odds ratios (95% CI) for preeclampsia (PE). Severe PE and small-for-gestational age (SGA) associated with maternal *FTO* SNP rs9939609 AA and AT genotypes as well as with each additional A allele (unadjusted and adjusted) in a Finnish study population.

	OR (95% CI) for outcome		
Covariates	PE	Severe PE**	SGA
TT	reference	reference	reference
AT*	1.30 (0.97.1.72)	1.31 (0.95.1.79)	1.05 (0.70.1.57)
AA*	1.12 (0.75.1.66)	0.98 (0.62.1.53)	0.83 (0.45.1.50)
Per A-allele, unadjusted*	1.10 (0.91.1.33)	1.05 (0.85.1.30)	0.94 (0.72.1.24)
Per A-allele, adjusted for log-transformed BMI*	1.09 (0.89.1.32)	1.04 (0.84.1.28)	0.91 (0.69.1.21)
Per A-allele, adjusted for log-transformed BMI, age and diabetes*	1.09 (0.90.1.32)	1.03 (0.83.1.28)	0.90 (0.68.1.20)

*Binary logistic regression analysis.

**Nonsevere PE cases excluded.
