# Erratum to “An Obesity-Related *FTO* Variant and the Risk of Preeclampsia in a Finnish Study Population”

**DOI:** 10.1155/2012/540682

**Published:** 2012-07-24

**Authors:** Miira Klemetti, Leena M. Hiltunen, Sanna Heino, Seppo Heinonen, Eero Kajantie, Hannele Laivuori

**Affiliations:** ^1^Department of Obstetrics and Gynecology, Helsinki University Central Hospital, P.O. Box 140, 00029 Helsinki, Finland; ^2^Department of Medical Genetics, Haartman Institute, Biomedicum Helsinki, University of Helsinki, P.O. Box 63, 00014 Helsinki, Finland; ^3^Women's Health Research Program, Faculty of Medicine, P.O. Box 20, University of Helsinki, 00014 Helsinki, Finland; ^4^Department of Hemostasis, Finnish Red Cross Blood Service, Kivihaantie 7, 00310 Helsinki, Finland; ^5^Department of Obstetrics and Gynecology, Kuopio University Hospital, P.O. Box 1777, 70211 Kuopio, Finland; ^6^Diabetes Prevention Unit, National Institute of Health and Welfare, P.O. Box 30, 00271 Helsinki, Finland; ^7^Hospital for Children and Adolescents, Helsinki University Central Hospital, P.O. Box 281, 00029 Helsinki, Finland

The original paper was published with an error in the BMI data. This regrettable error was revealed in late 2011 when information was retrieved on the study subjects of Data Set 2 for a new study from the original data collection sheets. The error has occurred years ago in the data entry phase. We have now recalculated all BMI-related analyses included in this paper. Because of the skewed distribution of the BMI, log transformation of the BMI was used in all analyses. All in all, the corrections made, listed in detail below, result in only minor changes in the numbers. As before, no clear association was observed between the* FTO* genotype and BMI in this study population. The only change worth mentioning is that, using the corrected data, BMI ≥ 30 kg/m^2^ was more common among the subjects with the AA genotype compared with those with the TT genotype (*P* = 0.044). Regarding this finding, we have added one sentence at the end of the second paragraph of the Discussion section. The finding does not change the conclusions of our study.

The following corrections were required.

**(1) Page 3, Methods, 2.5 Statistical Analyses, Second Paragraph**

The first sentence should read “Differences of the background characteristics between cases and controls were analyzed using the Student's *t*-test (continuous variables), and the Chi-square test or the Fisher's exact test (frequencies for discrete variables).”

**(2) Page 3, Results, 3.1 Characteristics of the Study Subjects, First Paragraph**

The third sentence should read “Expectably, the cases had a higher mean BMI than the controls.”

**(3) Page 3, Results, 3.1 Characteristics of the Study Subjects, Second Paragraph**

The second sentence should read “The mean BMI values among the PE cases in Data Sets 1, 2 and 3 were 24.3 kg/m^2^, 23.1 kg/m^2^ and 24.8 kg/m^2^, respectively, with the differences between Data Set 2 and the other two data sets being statistically significant (*P* = 0.047 and *P* = 0.009 for Data Set 2 versus Data Sets 1 and 3, resp.).”

**(4) Page 3, Results, 3.2 Risk Factors of Preeclampsia, First Paragraph**

The first sentence should read “Well-established risk factors of PE such as BMI (Odds Ratio [OR] for each increase of 10% in BMI = 1.26; 95% CI: 1.16, 1.37; *P* < 0.001), primiparity (OR = 1.37; 95% CI: 1.05, 1.79; *P* = 0.020) and pre-gestational diabetes (OR = 6.16; 95% CI: 1.38, 27.4; *P* = 0.017) were associated PE also in this study population when all three data sets were combined.”

The second sentence should read “When the data sets were analyzed separately, pre-pregnancy BMI was associated with PE in Data Set 1 (OR for each 10% higher BMI = 1.26, 95% CI: 1.14, 1.40; *P* < 0.001) but not in Data Set 2 (OR = 1.20; 95% CI: 0.99, 1.46; *P* = 0.059).”

The third sentence should read “However, pre-pregnancy BMI was associated with severe PE in both Data Set 1 (OR: 1.25; 95% CI: 1.12, 1.40; *P* < 0.001) and Data Set 2 (OR: 1.22; 95% CI: 1.01, 1.48, *P* = 0.039).”

**(5) Page 3, Results, 3.2 Risk Factors of Preeclampsia**

The second paragraph should read “Overall, 25.8% of all study subjects were overweight and 7.7% were obese. In Data Sets 1, 2 and 3, the percentages of overweight and obese patients were 26.0% and 7.0%, 16.7% and 4.5%, and 42.0% and 16.8%, respectively. No difference in the frequency of overweight was found between subjects homozygous for the A-allele of rs9939609 and those homozygous for the T-allele (*P* = 0.390). However, obesity was more common among the subjects with the AA genotype compared with those with the TT genotype (*P* = 0.044). In the entire study population, each additional A-allele corresponded to an increase in BMI of 1.5% (95% CI: 1.0%, 3.1%; *P* = 0.051). Among the cases and the controls, the increase in BMI per A allele was 1.9% (95% CI: −0.5%, 4.3%; *P* = 0.112) and 0.9% (95% CI: −1.0%, 2.7%; *P* = 0.360), respectively. In Data Sets 1, 2 and 3 the BMI increased 0.8% (95% CI: −1.1%, 2.7%; *P* = 0.416), 2.3% (95% CI: −0.2%, 4.8%; *P* = 0.075) and 5.2% (95% CI: −0.2%, 10.6%; *P* = 0.059) per each additional A-allele, respectively. The mean BMIs for the genotypes TT, AT and AA were 23.2, 23.5 and 23.5 for Data Set 1, 22.1, 22.8 and 23.1 for Data Set 2 and 24.2, 24.9 and 27.5 for Data set 3, respectively, with all differences between groups being statistically non-significant.”

**(6) Page 4, Table 1, Second Row**

The *N*, mean BMI (kg/m^2^) and SD values in the second row of the table should read as follows.

Primiparous subjects:

- PE cases: 302, 23.8, 4.5, respectively;
- Controls: 264, 22.2, 3.1, respectively;
- *P*-value: *P* < 0.001.

Multiparous subjects:

- PE cases: 162, 24.6, 4.4, respectively;
- Controls: 185, 23.2, 3.7, respectively;
- *P*-value: 0.001.

**(7) Page 5, Table 3, Fourth Row**

The OR values and the 95% confidence intervals “per A allele, adjusted for log-transformed BMI” in the fourth row of the table should read as follows:

 PE: 1.06 (0.87–1.29);
 severe PE: 1.01 (0.81, 1.25);
 SGA: 0.94 (0.71, 1.25).

**(8) Page 5, Table 3, Fifth Row**

The OR values and the 95% confidence intervals “per A allele, adjusted for log-transformed BMI, age and diabetes” in the fifth row of the table should read as follows:

 PE: 1.07 (0.88–1.30);
 severe PE: 1.01 (0.81, 1.25);
 SGA: 0.93 (0.70, 1.24).

**(9) Page 5, Discussion, Second Paragraph, Last Sentence**

The following last sentence was added to the second paragraph: “We could not show a clear association between the *FTO* genotype and BMI in this study population, although the frequency of obesity was higher among the subjects with the AA genotype compared with those with the TT genotype.”

**(10) Page 6, Discussion, Fifth Paragraph**

The first sentence should read “The present study population was relatively lean, with the mean BMIs ranging from 22.7 to 24.8 kg/m^2^ in the three Data Sets analysed.”

